# Listeria tempestatis sp. nov. and Listeria rocourtiae subsp. hofi subsp. nov.

**DOI:** 10.1099/ijsem.0.006774

**Published:** 2025-05-12

**Authors:** Phillip Brown, Alexandra Moura, Guillaume Valès, Nathalie Tessaud-Rita, Jefffrey Niedermeyer, Cameron Parsons, Alexandre Leclercq, Angela Harris, Ryan E. Emanuel, Sophia Kathariou, Marc Lecuit

**Affiliations:** 1 Department of Plant and Microbial Biology, North Carolina State University, Raleigh, North Carolina, USA; 2 Institut Pasteur, Université Paris Cité, Listeria National Reference Center and WHO Collaborating Center, Paris, France; 3 Institut Pasteur, Université Paris Cité, Inserm U1117, Biology of Infection Unit, Paris, France; 4 Department of Food, Bioprocessing and Nutrition Sciences, North Carolina State University, Raleigh, North Carolina, USA; 5 Department of Civil, Construction and Environmental Engineering, North Carolina State University, Raleigh, North Carolina, USA; 6 Nicholas School of the Environment, Durham, North Carolina, USA; 7 Necker-Enfants Malades University Hospital, Division of Infectious Diseases and Tropical Medicine, APHP, Institut Imagine, Paris, France

**Keywords:** average nucleotide identity (ANI), Firmicutes, *in silico* DNA–DNA hybridization (*is*DDH), *Listeria*, new taxa, whole-genome sequencing

## Abstract

In September 2018, Hurricane Florence resulted in major flooding in North Carolina, USA. Efforts to isolate *Listeria monocytogenes* and other *Listeria* spp. from Hurricane Florence floodwaters repeatedly yielded non-haemolytic *Listeria*-like isolates that could not be readily assigned to known *Listeria* taxa. Whole-genome sequence analyses against the 28 currently known *Listeria* species confirmed that the isolates constitute two new taxa within the genus *Listeria*. Taxon I, with one isolate, showed the highest similarity to *Listeria goaensis,* with an average nucleotide identity blast of 85.3±4.4% and an *in silico* DNA–DNA hybridization (*is*DDH) of 32.4% (range: 30–35%), differing from the latter by its ability to reduce nitrate, ferment d-ribose and sucrose, and by its inability to produce catalase or ferment d-trehalose and d-lactose. Taxon II, represented by 11 isolates, showed the highest similarity to *Listeria rocourtiae,* with an average nucleotide identity blast of 92.64±3.8% and an *is*DDH of 49.9% (range: 47.3–52.5%), differing from the latter by its ability to ferment l-arabinose and its inability to ferment l-rhamnose, d-galactose, d-lactose and d-melibiose. The names *Listeria tempestatis* sp. nov. and *Listeria rocourtiae* subsp. *hofi* subsp. nov. are proposed for taxon I and II, respectively, with type strains CLIP 2022/01175^T^ (F6L-1A=CIP 112444^T^ = DSM 117029^T^) and CLIP 2022/01000^T^ (F66L-1A=CIP 112443^T^ = DSM 117030^T^), respectively. Both taxa lack known *Listeria* pathogenic islands, suggesting a lack of pathogenicity for humans.

## Introduction

The genus *Listeria* belongs to the phylum *Bacillota*, consisting of Gram-positive, rod-shaped bacteria that are typically catalase positive, oxidase negative and have a low G+C content [1]. Currently, 28 *Listeria* species have been described, with 22 identified only since 2010, largely due to increased efforts to isolate *Listeria* from diverse sources, especially aquatic ecosystems, the increased availability of whole-genome sequencing and the use of whole-genome sequence data in taxonomic classification. The genus *Listeria* can be partitioned into two major clades [2,3]: (*i*) *Listeria sensu stricto*, with ten species (*L. monocytogenes* [4], *L. innocua* [5], *L. welshimeri* [6], *L. seeligeri* [6], *L. ivanovii* [7], *L. marthii* [8], *L. cossartiae* [9], *L. farberi* [9]*, L. immobilis* [9] and ‘*L. swaminathanii’* [10]) and (*ii*) *Listeria sensu lato*, containing 18 species (*L. grayi* [11], *L. rocourtiae* [12], *L. weihenstephanensis* [13]*, L. aquatica* [14]*, L. cornellensis* [14]*, L. fleishchmannii* [14]*, L. floridensis* [14], *L. grandensis* [14], *L. riparia* [14]*, L. booriae* [15]*, L. newyorkensis* [15], *L. costaricensis* [16], *L. goaensis* [17]*, L. thailandensis* [18], *L. valentina* [19], *L. rustica* [9]*, L. portnoyi* [9] and *L. ilorinensis* [20]). All species are non-pathogenic except for *L. monocytogenes,* which is considered the only human pathogen in the genus *Listeria,* and *L. ivanovii,* which predominantly infects ruminants and, rarely, humans [21]. *L. monocytogenes* represents an important concern for food safety and public health due to the severe symptoms and high case-fatality and hospitalization rates of listeriosis [2,22].

In September 2018, Hurricane Florence caused massive flooding of many inland rivers and other locations in eastern North Carolina, USA, a largely rural region highly dense in facilities producing and processing food animals, especially swine and poultry [23,24]. Samples from Hurricane Florence floodwaters were collected and analysed for *L. monocytogenes* and other *Listeria* species, as well as other bacterial pathogens [25,26]. Several *Listeria*-like isolates that could not be readily assigned to published *Listeria* taxa were identified. Here, genotypic and phenotypic analyses were conducted to designate appropriate taxonomic classifications for 12 of these isolates.

## Methods

### Bacterial strains and growth conditions

Unless stated otherwise, all isolates were grown in brain heart infusion (BHI) at 37 °C for 24 h, and strains used as controls were * L. booriae* CLIP 2016/00711, *L. ivanovii* subsp. *ivanovii* CLIP 12510^T^, *L. ivanovii* subsp. *londonensis* CLIP 12229^T^, *L. valentina* CLIP 2019/00642^T^ (DSM 110544^T^), *L. thailandensis* CLIP 2015/00305^T^ (DSM 107638^T^) and/or *L. monocytogenes* CLIP 74910 (ATCC 19115), as described below. *L. rocourtiae* CIP 109804^T^ and *L. goaensis* CLIP 2022/00569^T^ (KCTC 33909^T^), the closest species to the new taxa, were included in all experiments.

Floodwater samples were analysed for *Listeria* spp. as previously described [27]. Briefly, for primary enrichments, 1.25 ml of the water samples were mixed with 11.25 ml of half Fraser broth supplemented with half Fraser selective supplement (Oxoid, Hampshire, UK) and incubated at 30 °C for 24–48 h. Secondary enrichments consisted of 100 µl of the primary enrichment in 10 ml of full Fraser broth with full Fraser selective supplement (Oxoid) and were incubated at 37 °C for 48 h. The primary and secondary enrichments (20 µl) were plated on modified Oxford medium (MOX; Becton, Dickinson and Co., Sparks, MD, USA) and incubated at 37 °C for 48 h. Colonies typical of *Listeria* spp. (round, black in colour, smooth, flat or slightly convex in shape) were purified on blood agar plates (trypticase soy agar with 5% sheep blood; Remel, San Diego, CA, USA) and analysed via multiplex PCR to determine tentative serogroup designations as described [28,29]. Twelve *Listeria*-like isolates that failed to yield an amplicon were sent to the World Health Organization Collaborating Centre *Listeria* (Institut Pasteur, Paris, France) for identification and further characterization.

### Species identification, genome sequencing and analysis

Initial efforts for species identification employed the MALDI-TOF mass spectrometry Sirius system and the MBT library version 12.0.0.0 (Bruker Daltonics, Bremen, Germany) [30]. For DNA sequence-based identification, genomic DNA was extracted using the NucleoSpin Tissue purification kit (Macherey-Nagel, Düren, Germany) from 0.9 ml of cultures grown overnight in brain heart infusion (BHI, Difco, France) at 35 °C. DNA libraries were prepared using the Nextera XT DNA Sample kit (Illumina, California, USA) and sequenced with Illumina NextSeq 500 using a 2×150 bp paired-end run. Raw reads were trimmed with fqCleanER v.21.10 (https://gitlab.pasteur.fr/GIPhy/fqCleanER) as previously described [31] and assembled with SPAdes v.3.14 [32] with the automatic k-*mer*, --only-assembler and --careful options. Contigs longer than 300 bp were annotated with Prokka v.1.14 [33], and screening for plasmids utilized MOB-suite v.2.0.1 [34]. In addition, the 16S rRNA gene was amplified with primers 27F (5′-AGAGTTTGATCMTGGCTCAG-3′) and 1492R (5′-GGTTACCTTGTTACGACTT-3′) [35] and amplicons were Sanger sequenced.

Phylogenetic analyses were performed based on the nucleotide sequences of the 16S rRNA genes and on the core genome alignment of all *Listeria* species, defined using Roary v.3.13 [36] and a BLASTP identity cut-off of 80% to identify orthologs [20]. Maximum likelihood phylogenetic trees were constructed using IQ-Tree v.2.2 [37], and the best fit substitution models (TPM3u+F+I+G4 for 16S rRNA and GTR+F+I+G4 for the core genome) were identified using ModelFinder [38] and visualized in mega v.7.0 [39]. The average nucleotide identity (ANIb) shared with other *Listeria* species was determined using the enveomics package [40], with the blastn settings defined as in JSpecies v1.2.1 and Goris *et al.* [41]. Dendrograms based on the unweighted pair group method with arithmetic mean (UPGMA) method were obtained from the distance matrices using BioNumerics v.7.6 (Applied Maths, Belgium). The average *in silico* DNA–DNA hybridization (*is*DDH) similarities were calculated using the GGDC 2.1 web server and formula 2 [sum of all identities found in high-scoring segment pairs (HSP) divided by overall HSP length] [42]. *In silico* PCR-serogrouping profiles were obtained from the draft genomes as described previously [28,36].

### Phenotypic characterization

Gram staining was performed with the Color Gram 2 kit (bioMérieux, Marcy I’Etoile, France), according to the manufacturer’s instructions. Catalase and oxidase activities were determined using the API ID Color Catalase kit (bioMérieux) and the Bactident Oxidase test strips (Merck Millipore, France), respectively, according to the manufacturer’s instructions. Respiratory characteristics were determined in tubes containing meat liver agar (Bio-Rad, France) at 30 °C after 24 h, following the manufacturer’s instructions. The presence of a capsule was assessed using India ink staining. Growth characteristics were determined on BHI agar and broth at 4 °C for 10 days and at 22, 37 and 42 °C for 7 days. Growth was considered positive if there was an increase in cell number of at least 1.0 log (c.f.u./ml^−1^). Isolates were grown on Rapid’*L.mono* agar (Bio-Rad) and Agar *Listeria* according to Ottaviani and Agosti (ALOA; bioMérieux) plates at 30 and 37 °C for 24 h.

Motility was tested by stab inoculation in U-shaped glass tubes containing tryptic soy semi-solid agar (Bio-Rad), followed by incubation at 22 and 37 °C for 7 days in aerobic conditions. Haemolytic activity was assessed both by growth on sheep blood agar plates as described above and by stabbing the isolates into Columbia agar plates (bioMérieux) containing 5% defibrinated horse blood, as described in the *Bacteriological Analytical Manual* [43], followed by incubation for 24 h at 37 °C. The Christie, Atkins, Munch-Petersen (CAMP) test was performed as previously described, by streaking isolates and controls horizontally on Columbia agar containing 5% defibrinated sheep blood (bioMérieux), together with *Rhodococcus equi* NCTC 1621, which was streaked vertically. *L. ivanovii* was used as a positive control, while *L. rocourtiae* and *L. goaensis* were used as negative controls. Plates were incubated at 37 °C for 24 h and examined for enhanced haemolysis at the confluence of the horizontal and vertical streaks.

Nitrate reduction was determined by inoculation in nitrate broth (bioMérieux) and incubation at 37 °C for 5 days, with *L. rocourtiae* used as a positive control, while *L. goaensis* and *L. monocytogenes* were used as negative controls. Carbohydrate fermentation profiles were determined with API *Listeria* and the API50CH system (bioMérieux), following the manufacturer’s recommendations, using *L. monocytogenes, L. rocourtiae* and *L. goaensis* as controls and following incubation at 37 °C for 24 h. The production of acetoin from glucose fermentation (Voges-Proskauer test) was assessed using API20E strips (bioMérieux) and recorded after incubation at 37 °C for 24 h. *L. monocytogenes*, *L. booriae*, *L. rocourtiae* and *L. goaensis* were used as controls.

### Antimicrobial susceptibility

Susceptibility to antimicrobials was determined with the disc diffusion method on Mueller–Hinton agar plates (Bio-Rad) at 30 °C, following the recommendations from the European Committee on Antibiotic Susceptibility Testing (EUCAST, https://www.eucast.org/). The following antibiotic discs (Bio-Rad) were used: amoxicillin (25 µg), ampicillin (10 µg), cefotaxime (30 µg), chloramphenicol (30 µg), ciprofloxacin (5 µg), clindamycin (2 µg), erythromycin (15 µg), fosfomycin (50 µg), fusidic acid (10 µg), gentamicin (15 µg), imipenem (10 µg), kanamycin (30 µg), levofloxacin (5 µg), penicillin G (6 µg), moxifloxacin (5 µg), nalidixic acid (30 µg), rifampicin (30 µg), streptomycin (10 µg), sulphonamides (200 µg), tetracycline (30 µg), trimethoprim (5 µg) and vancomycin (30 µg). The diameters of inhibition zones were measured with the automatic reader Scan 4000 (Interscience, France).

## Results and discussion

### MALDI-TOF identification

Species identification efforts using the MALDI-TOF mass spectrometry Sirius system and the MBT library version 12.0.0.0 [30] were inconclusive for nine isolates (scores <2.00, indicative of *Listeria* spp.) and indicative of *L. newyorkensis* for three isolates (scores 2.02–2.05).

### Genome sequence and analyses

Sequence metrics of the 12 isolates (Table 1) met the quality standards for taxonomic determination [44]. Draft assemblies ranged between 2.8 and 3.4 Mb and had a G+C content between 38.6 and 40.6 mol% (Table 1), similar to other species of the genus *Listeria* (2.6 and 3.5 Mb, 35.9 and 43.6 mol% G+C). No plasmids were detected in any of the 12 isolates. Sequence-based serogrouping by analysis of the multiplex PCR target sequences revealed the *prs* gene (serogroup L, typical of non-*L. monocytogenes* species and certain uncommon *L. monocytogenes* serotypes) in both new taxa. The 16S rRNA gene sequence similarity of the two taxa (Suppl. Fig. 1, available in the online version of this article) was 100% with *L. rocourtiae* and 99.4% with *L. fleischmannii,* thus above the proposed cut-off of 98.7–99.0% [45], below which strains do not belong to the same species, and confirming the lack of adequate resolution of 16S rRNA gene sequence analysis for species delimitation.

**Table 1.** Genome metrics of isolates obtained in this study

Isolate*	Alias	Geographic location	Geographic coordinates	Isolation date	No. of reads	Coverage (x)	No. of contigs	Total length (bp)	N50	GC%	Closest species	ANIb with closest species	*is*DDH with closest species	SRA accession no.	BIGSdb id
*L. tempestatis* sp. nov.															
CLIP 2022/01775^T^	F6L-1A	Grifton, NC, USA	35.38 N, −77.45 W	2018-09-21	5.94E+06	318	38	2.83E+06	3.04E+05	38.6	*L. goaensis*	85.3±4.36%	32.4% (30.0–35.0%)	ERR12145462	98880
*L. rocourtiae* subsp. *hofi* subsp. nov.															
CLIP 2022/01000^T^	F66L-1A	Fairmont, NC, USA	34.55 N, −79.19 W	2018-10-18	9.58E+06	449	124	3.28E+06	6.60E+04	40.5	*L. rocourtiae*	92.1±4.8%	49.9% (47.3–52.5%)	ERR12145451	84502
CLIP 2022/01784	F61L-2A	Lumberton, NC, USA	34.62 N, −79.01 W	2018-10-18	5.92E+06	277	112	3.24E+06	6.66E+04	40.6	*L. rocourtiae*	92.0±4.92%	49.5% (46.9–52.2%)	ERR12145452	98889
CLIP 2022/01786	F67L-1A	Fairmont, NC, USA	34.53 N, −79.17 W	2018-10-18	6.18E+06	290	111	3.30E+06	6.72E+04	40.6	*L. rocourtiae*	92.1±4.83%	49.4% (46.7–52.0%)	ERR12145453	98891
CLIP 2022/01787	F67L-2A	Fairmont, NC, USA	34.53 N, −79.17 W	2018-10-18	5.62E+06	263	104	3.23E+06	7.07E+04	40.5	*L. rocourtiae*	92.2±4.53%	49.9% (47.3–52.6%)	ERR12145454	98892
CLIP 2022/01789	F70L-1A	Goldsboro, NC, USA	35.38 N, −77.82 W	2018-10-18	5.52E+06	259	104	3.34E+06	7.33E+04	40.6	*L. rocourtiae*	92.1±4.86%	49.4% (46.7–52.0%)	ERR12145455	98894
CLIP 2022/01790	F70L-2A	Goldsboro, NC, USA	35.38 N, −77.82 W	2018-10-18	6.08E+06	285	352	3.36E+06	7.47E+04	40.5	*L. rocourtiae*	92.2±4.47%	49.9% (47.3–52.6%)	ERR12145456	98895
CLIP 2022/01795	F79L-2A	Goldsboro, NC, USA	35.38 N, −77.87 W	2018-10-18	5.92E+06	277	115	3.30E+06	7.34E+04	40.6	*L. rocourtiae*	92.0±4.93%	49.4% (46.8–52.0%)	ERR12145457	98900
CLIP 2022/01796	F84L-1A	Goldsboro, NC, USA	35.38 N, −77.82 W	2018-10-18	6.51E+06	305	101	3.29E+06	7.41E+04	40.6	*L. rocourtiae*	92.0±4.86%	49.4% (46.8–52.0%)	ERR12145458	98901
CLIP 2022/01797	F86L-2A	Piney Green, NC, USA	35.11 N, −78.48 W	2018-10-18	6.25E+06	293	103	3.23E+06	7.83E+04	40.5	*L. rocourtiae*	92.3±4.46%	50.0% (47.3–52.6%)	ERR12145459	98902
CLIP 2022/01798	F89L-2A	Comfort, NC, USA	35.00 N, −77.48 W	2018-10-18	6.43E+06	301	112	3.30E+06	6.72E+04	40.6	*L. rocourtiae*	92.0±4.91%	49.4% (46.8–52.0%)	ERR12145460	98903
CLIP 2022/01799	F101L-2A	Northwest, NC, USA	34.32 N, −78.18 W	2018-10-19	4.45E+06	208	110	3.29E+06	6.62E+04	40.6	*L. rocourtiae*	92.0±4.90%	49.4% (46.8–52.0%)	ERR12145461	98904

*CLIP, Collection *Listeria* Institut Pasteur.

**Fig. 1.**
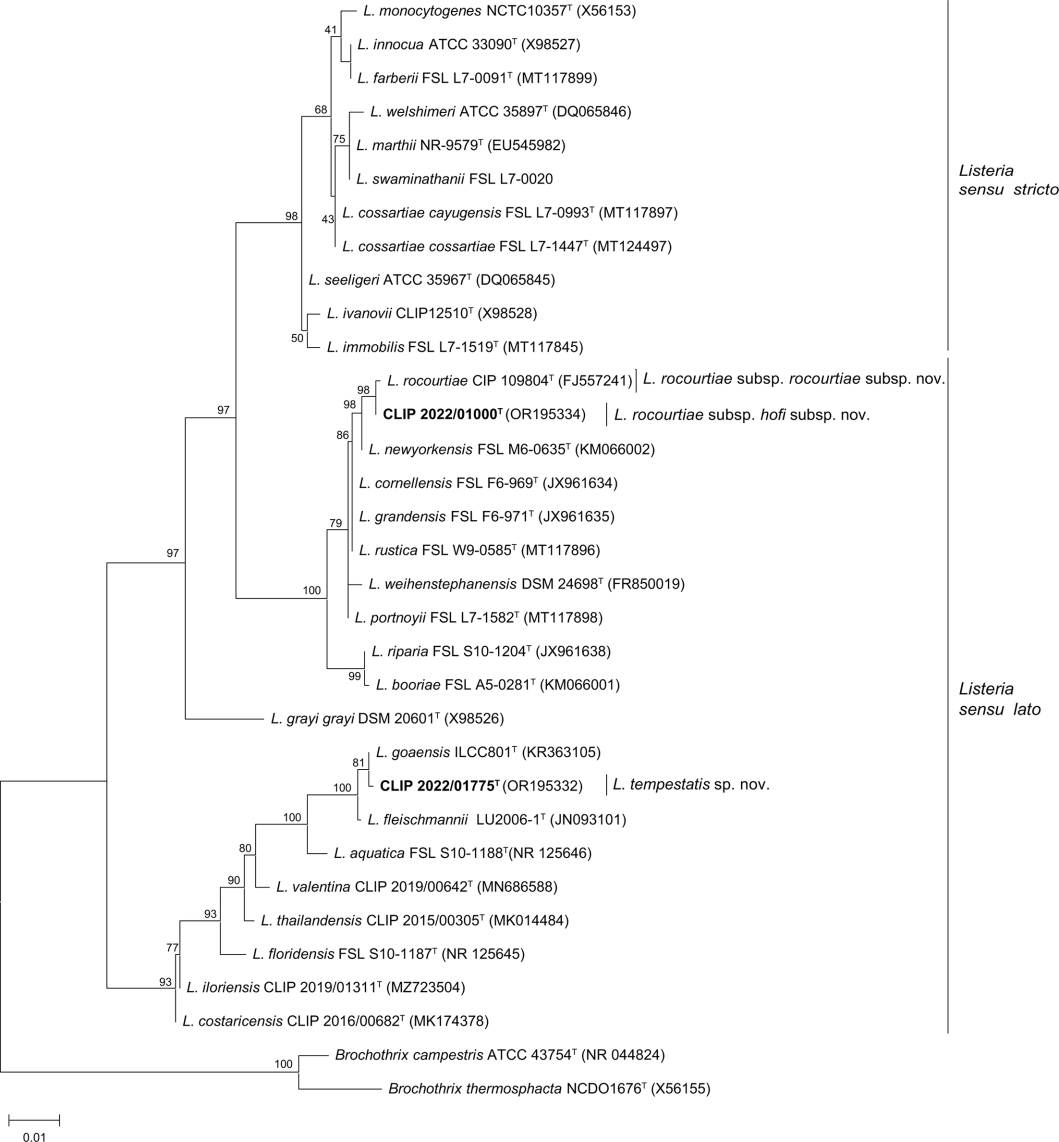
Maximum likelihood phylogenetic analysis based on the core gene alignment of 35 *Listeria* reference strains and the 12 isolates of this study. Distance estimation was obtained by the GTR+F+I+G4 model [38], based on an alignment of 80,539 positions. Branch lengths represent the number of nucleotide substitutions per site, and bootstrap percentages of 1,000 replicates are shown. GenBank accession numbers are provided in brackets. *Listeria tempestatis* sp. nov. and *Listeria rocourtiae* subsp. *hofi* subsp. nov. are highlighted in bold.

Core genome analyses based on the 305 genes present in 95–100% of *Listeria* species representatives (Fig. 1) showed the highest sequence similarity of the two taxa with *L. rocourtiae* and *L. goaensis*. Isolates shared less than 95% ANIb and 70% *is*DDH (Table 1 and Suppl. Fig. 2), the proposed genomic cut-offs for species identification [44], with all known species. The mostly closely related species to the first taxon (11 isolates: CLIP 2022/01000^T^, CLIP 2022/01784, CLIP 2022/01786, CLIP 2022/01787, CLIP 2022/01789, CLIP 2022/01790, CLIP 2022/01795, CLIP 2022/01796, CLIP 2022/01797, CLIP 2022/01798 and CLIP 2022/01799) with a validly published name was *L. rocourtiae* (CLIP 2022/01000^T^: two-way ANIb of 92.10±4.78%, based on 14,248 genome fragments; *is*DDH of 49.9% [47.3–52.5%]) and the most closely related species to the second taxon (one isolate: CLIP 2022/01775^T^) was *L. goaensis* (CLIP 2022/01775^T^: two-way ANIb of 85.26±4.36% based on 12,669 genome fragments; *is*DDH of 32.4% [30.0–35.0%]). These results confirmed that the 12 isolates constitute two distinct novel taxa within the *Listeria* genus, for which the names *Listeria rocourtiae* subsp*. hofi* subsp. nov. (11 isolates) and *Listeria tempestatis* sp. nov. (1 isolate) are proposed.

### Phenotypic characterisation

*L. tempestatis* sp. nov. and all isolates of *L. rocourtiae* subsp*. hofi* subsp. nov. were Gram-positive bacilli without endospores or capsules, facultative anaerobic and oxidase negative. On Rapid’*L.mono* agar, colonies were white, indicating no phosphatidylinositol-specific phospholipase C (PI-PLC) activity, surrounded by a yellow halo (d-xylose fermentation). On ALOA, colonies were blue (β-glucosidase activity) without halos (no PI-PLC activity).

Neither *L. tempestatis* sp. nov. nor any of the isolates of *L. rocourtiae* subsp*. hofi* subsp. nov. were motile at 22 or 37 °C, consistent with the absence of motility genes in their genomes*. L. tempestatis* sp. nov. and all isolates of *L. rocourtiae* subsp*. hofi* subsp. nov. were also negative for haemolysis and the CAMP tests, consistent with the absence of known *Listeria* pathogenicity islands within their draft genomes, as observed in all other *Listeria sensu lato* species.

Similarly to most *Listeria* species except *L. costaricensis* and *L. ilorinensis,* all isolates of *L. rocourtiae* subsp*. hofi* subsp. nov. were catalase positive. In contrast, and differing in this regard from most *Listeria* species, including its closest relative *L. goaensis*, *L. tempestatis* sp. nov. was catalase negative, consistent with the absence of catalase genes in its genome.

*L. tempestatis* sp. nov. was able to grow at all tested temperatures except at 4 °C, similarly to its closest species, *L. goaensis.* On the other hand, all isolates of *L. rocourtiae* subsp*. hofi* subsp. nov. were able to grow at all tested temperatures except at 42 °C, similarly to *L. rocourtiae* subsp. *rocourtiae* subsp. nov.

Biochemical characterization of *L. rocourtiae* subsp*. hofi* subsp. nov. and *L. tempestatis* sp. nov. is summarized in Suppl. Table 1. *L. tempestatis* sp. nov. was able to reduce nitrate, contrary to its closest species, *L. goaensis*. All isolates of *L. rocourtiae* subsp*. hofi* subsp. nov. were able to reduce nitrate, similarly to *L. rocourtiae* subsp. *rocourtiae* subsp. nov.

Carbohydrate fermentation profiles play an important role in *Listeria* characterization, as these profiles help differentiate between species based on metabolic capabilities. *L. tempestatis* sp. nov. was able to ferment amygdalin, arbutin, d-cellobiose, d-xylose, aesculin ferric citrate, d-fructose, gentiobiose, d-glucose, d-maltose, d-mannitol, methyl-α-d-glucopyranoside, d-mannose, *N*-acetylglucosamine, l-rhamnose, d-ribose, d-saccharose, salicin and xylitol. d-Arabitol fermentation was variable depending on substrate concentration [negative in API50CH (1.4 mg) but positive in API *Listeria* (0.4 mg); Suppl. Table 1].

All isolates of *L. rocourtiae* subsp*. hofi* subsp. nov. were able to ferment the following substrates: amygdalin, arbutin, d-cellobiose, d-xylose, aesculin ferric citrate, d-fructose, gentiobiose, d-glucose, l-arabinose, d-maltose (weak), methyl-α-d-glucopyranoside, d-mannose, *N*-acetylglucosamine, d-ribose, salicin and d-trehalose. Similarly to certain *Listeria sensu lato* species, including *L. booriae*, *L. rocourtiae* and *L. goaensis*, the Voges-Proskauer test was negative for *L. tempestatis* sp. nov. and for all isolates of *L. rocourtiae* subsp*. hofi* sp. nov.

UPGMA analysis of all species phenotypic traits also showed that *L. tempestatis* sp. nov. is closest to *L. goaensis*, differing from the latter only by its ability to ferment d-ribose and sucrose and its inability to ferment d-trehalose or d-lactose, as well as being positive for nitrate reductase and negative for catalase (Table S1). All isolates of *L. rocourtiae* subsp*. hofi* subsp. nov. differ from *L. rocourtiae* subsp. *rocourtiae* subsp. nov. by their ability to ferment l-arabinose and inability to ferment l-rhamnose, d-galactose, d-lactose and d-melibiose. These specific differences in carbohydrate metabolism are important species-differentiating factors and should be considered for precise identification in the absence of whole-genome sequencing.

*L. tempestatis* sp. nov. was sensitive to all tested antibiotics, except amoxicillin, ampicillin, cefotaxime and penicillin, which resulted in no detectable inhibition halo. All isolates of *L. rocourtiae* subsp*. hofi* subsp. nov. were sensitive to all tested antibiotics, except to clindamycin and fosfomycin, for which no inhibition halo was detected. Genes conferring resistance towards antibiotics or tolerance towards disinfectants were not detected. The observed resistance traits were shared with their closest respective species, suggestive of intrinsic resistance mechanisms as reported for these antibiotics in *L. monocytogenes* [46].

In conclusion, this study reports the discovery of two novel *Listeria* taxa, *L. tempestatis* sp. nov. and *L. rocourtiae* subsp. *hofi* subsp. nov., isolated from hurricane floodwaters, expanding our understanding of the *Listeria* genus. Continued investigations into *Listeria* diversity are crucial to identify characterize new taxa, especially from previously understudied sources such as the aquatic ecosystem. Such investigations can offer insights into *Listeria* evolution, ecology and adaptations and into potential impacts of this ubiquitous genus of bacteria on public health and food and water safety, particularly in the context of current global environmental changes.

## Description of *Listeria tempestatis* sp. nov.

*Listeria tempestatis* sp. nov. (tem.pes.ta'tis. L. gen. n. *tempestatis*, meaning ‘of a storm’; named after the hurricane floodwaters from which the strain was originally isolated).

Cells are straight, Gram-stain-positive, non-spore-forming and non-encapsulated short rods. Facultative anaerobic, catalase negative and oxidase negative. Colonies are opaque with a flat shape and entire margins on BHI. On ALOA, colonies exhibit blue colour (due to β-glucosidase activity) without a surrounding halo (lack of PI-PLC activity), typical of *Listeria* non-haemolytic species. Growth occurs at 22–42 °C, with optimal growth between 22 and 37 °C. Non-motile at both 22 and 37 °C. Negative for haemolysis and nitrite reduction but positive for nitrate reduction. Voges-Proskauer test negative. After 24 h, acid is produced from amygdalin, arbutin, d-cellobiose, d-xylose, aesculin ferric citrate, d-fructose, gentiobiose, d-glucose, d-maltose, methyl-α-d-glucopyranoside, d-mannose, *N*-acetylglucosamine, l-rhamnose, d-ribose, d-saccharose and salicin. Phenotypically, *L. tempestatis* sp. nov. can be differentiated from its current closest species, *L. goaensis*, by its ability to reduce nitrate and ferment d-ribose and sucrose, and its inability to produce catalase or ferment d-trehalose.

The type strain CLIP 2022/01775 (F6L-1A) was isolated on 18 October 2018 from floodwaters produced by Hurricane Florence in eastern North Carolina, USA. The genomic DNA G+C content of the type strain is 38.64 mol%. The type strain is deposited at the Leibniz Institute DSMZ – German Collection of Microorganisms (DSM 117029^T^) and the Collection of Institut Pasteur (CIP 112444^T^). The GenBank/EMBL/DDBJ accession numbers for the 16S rRNA gene sequence and the draft genome of the type strain are OR195332 and CAUYTK01, respectively.

## Description of *Listeria rocourtiae* subsp*. rocourtiae* subsp. nov.

*Listeria rocourtiae* subsp. *rocourtiae* subsp. nov. (ro.cour′ti.ae. N.L. fem. gen. n. *rocourtiae,* named after the French bacteriologist Jocelyne Rocourt).

General characteristics have been previously described [12]. Cells are Gram-stain-positive, non-spore-forming short rods. Facultative anaerobic, non-haemolytic, catalase positive, nitrate reduction positive and oxidase negative. Colonies are round with a low convex and entire margin on trypto-casein-soy agar when incubated at 30 °C. Growth occurs at 4–37 °C. After 24 h, acid is produced from l-galactose, d-glucose, glycerol, d-lactose, d-maltose, d-mannitol, d-melibiose, methyl α-d-glucopyranoside, l-rhamnose, d-ribose, d-xylose, *N*-acetylglucosamine, amygdalin, arbutin, d-cellobiose, d-fructose, d-mannose and salicin.

The type strain is strain CIP 109804^T^ (=DSM 22097^T^ =Allerberger 700284/02^T^).

## Description of *Listeria rocourtiae* subsp*. hofi* subsp. nov.

*Listeria rocourtiae* subsp*. hofi* subsp. nov. (ho’fi N.L. masc. n. *hofi*, named in honour of Dr Herbert Hof, for his research and other pioneering contributions on *Listeria monocytogenes* and listeriosis).

Cells are straight, Gram-stain-positive, non-spore-forming and non-encapsulated short rods. Facultative anaerobic, catalase positive and oxidase negative. Colonies are opaque with a flat shape and entire margins on BHI. On ALOA, colonies exhibit blue colour (due to β-glucosidase activity) without a surrounding halo (lack of PI-PLC activity), typical of non-haemolytic *Listeria* species. Growth occurs at 4–37 °C, with optimal growth at 30 °C. Non-motile at both 22 and 37 °C. Negative for haemolysis and nitrite reduction but positive for nitrate reduction. Voges-Proskauer test negative.

After 24 h, acid is produced from amygdalin, arbutin, d-cellobiose, d-xylose, aesculin ferric citrate, d-fructose, gentiobiose, d-glucose, l-arabinose, d-maltose (weak), methyl-α-d-glucopyranoside, d-mannose, *N*-acetylglucosamine, d-ribose, salicin and d-trehalose. d-Arabitol fermentation is variable depending on substrate concentration, being negative in API50CH (1.4 mg) but positive in API *Listeria* (0.4 mg). The production of acid from glycerol is variable among the isolates. Phenotypically, all isolates of *L. rocourtiae* subsp*. hofi* subsp. nov. can be differentiated from *L. rocourtiae* subsp. *rocourtiae* subsp. nov. by their ability to ferment l-arabinose and inability to ferment l-rhamnose, d-galactose, d-lactose and d-melibiose.

The type strain CLIP 2022/01000 (F66L-1A) was isolated on 18 October 2018 from floodwaters produced by Hurricane Florence in eastern North Carolina, USA. The genomic DNA G+C content of the type strain is 40.48 mol%. The type strain is deposited at the Leibniz Institute DSMZ – German Collection of Microorganisms (DSM 117030^T^) and the Collection of Institut Pasteur (CIP 112443^T^). The GenBank/EMBL/DDBJ accession numbers for the 16S rRNA gene sequence and draft genome of the type strain are OR195334 and CAUYTL01, respectively.

## Supplementary material

10.1099/ijsem.0.006774 Uncited Supplementary Material 1.

## Abbreviations

AAI: average amino acid identity
ANI: average nucleotide identity
BHI: brain heart infusion
BLAST: basic local alignment search tool
CAMP: Christie, Atkins, Munch-Petersen test
CIP: Collection of Institut Pasteur
CLIP: Collection *Listeria* Institut Pasteur
DDBJ: DNA Data Bank of Japan
DSMZ: German Collection of Microorganisms and Cell Cultures
EMBL: European Molecular Biology Laboratory
isDDH: *in silico* DNA–DNA hybridization
N50: minimum contig length covering 50% of the genome
PCR: polymerase chain reaction
PI-PLC: phosphatidylinositol-specific phospholipase C
UPGMA: unweighted pair group method with arithmetic mean
