# Supplementary material for: Listeria tempestatis sp. nov. and Listeria rocourtiae subsp. hofi subsp. nov
Source: Int J Syst Evol Microbiol. 2025 May 12;75(5):006774. doi: 10.1099/ijsem.0.006774 (PMC12378429; doi:10.1099/ijsem.0.006774)
Supplement: Uncited Supplementary Material 1. [file ijsem-75-06774-s001.pdf]

Supplementary Material

Listeria tempestatis sp. nov. and *Listeria rocourtiae* subsp. *hofi* subsp. nov. isolated from hurricane floodwaters in North Carolina, USA

Phillip Brown[1*], Alexandra Moura[2,3*], Guillaume Valès[2], Nathalie Tessaud-Rita[2], Jeffrey Niedermeyer[4], Cameron Parsons[4], Alexandre Leclercq[2], Angela Harris[5], Ryan E. Emanuel[6], Sophia Kathariou[1,4#], Marc Lecuit[2,3,7,#]

[1]Department of Plant and Microbial Biology, North Carolina State University, Raleigh, North Carolina, USA

[2]Institut Pasteur, Université Paris Cité, National Reference Center and WHO Collaborating Center *Listeria*, Paris, France

[3]Institut Pasteur, Université Paris Cité, Inserm U1117, Biology of Infection Unit, Paris, France

[4]Department of Food, Bioprocessing and Nutrition Sciences, North Carolina State University, Raleigh, North Carolina, USA

[5]Department of Civil, Construction and Environmental Engineering, North Carolina State University, Raleigh, North Carolina, USA

[6]Nicholas School of the Environment, Durham, North Carolina, USA

[7]Necker-Enfants Malades University Hospital, Division of Infectious Diseases and Tropical Medicine, APHP, Institut Imagine, Paris, France

* These authors share co-first authorship.

[#] To whom correspondence should be addressed: marc.lecuit@pasteur.fr & skathar@ncsu.edu

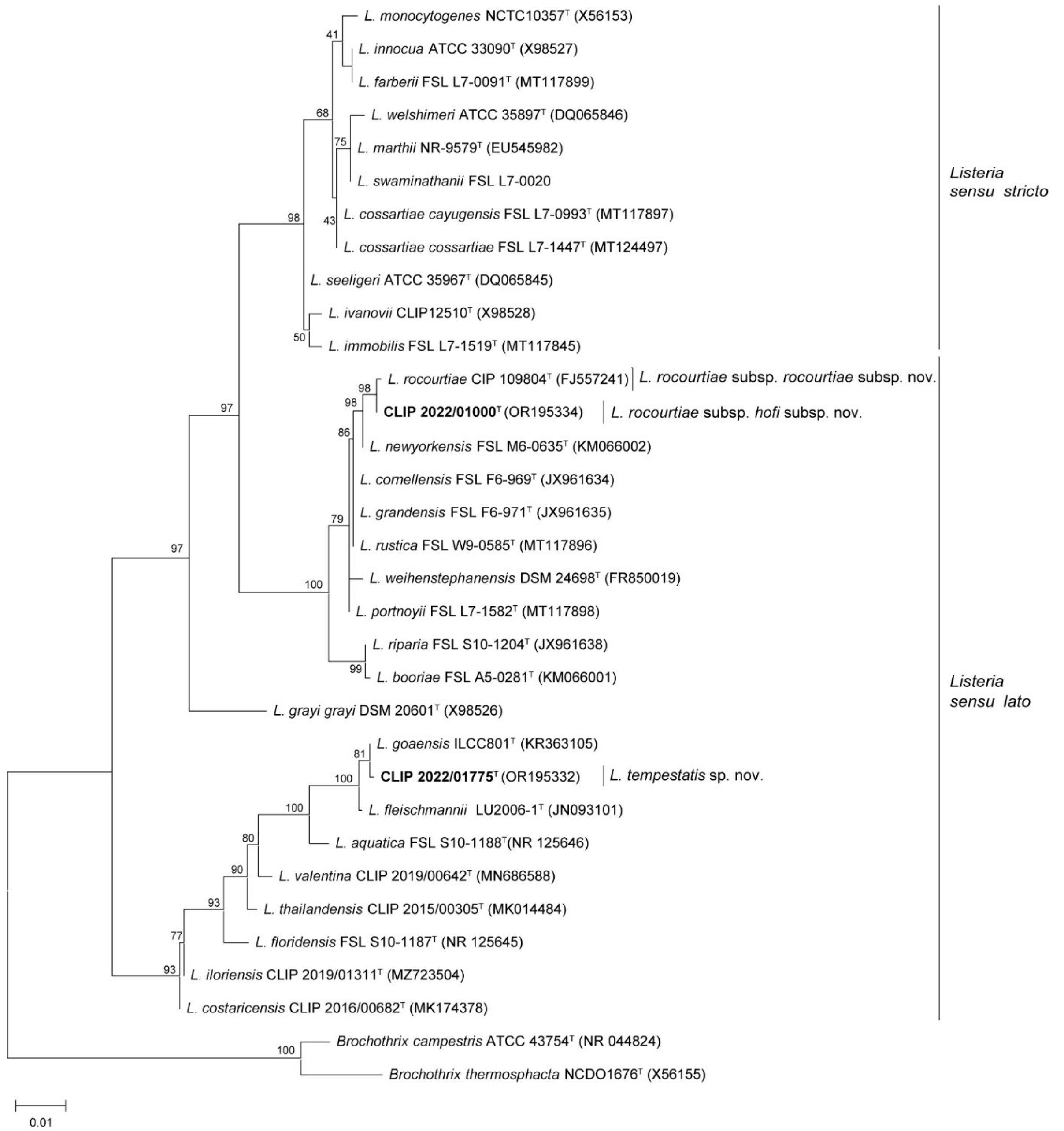

Supplementary Figure 1. Maximum likelihood phylogeny based on 16S rRNA gene sequences. Distance estimations were obtained by the model of TPM3u+F+I+G4 (38), based on an alignment of 1,116 positions. Selected members of the genus *Brochothrix* were used as the outgroup. Branch lengths represent the number of nucleotide substitutions per site and bootstrap percentages of 1,000 replicates are shown. GenBank accession numbers are provided in brackets. *Listeria tempestatis* sp. nov. and *Listeria rocourtiae* subsp. *hofi* subsp. nov. are highlighted in bold.

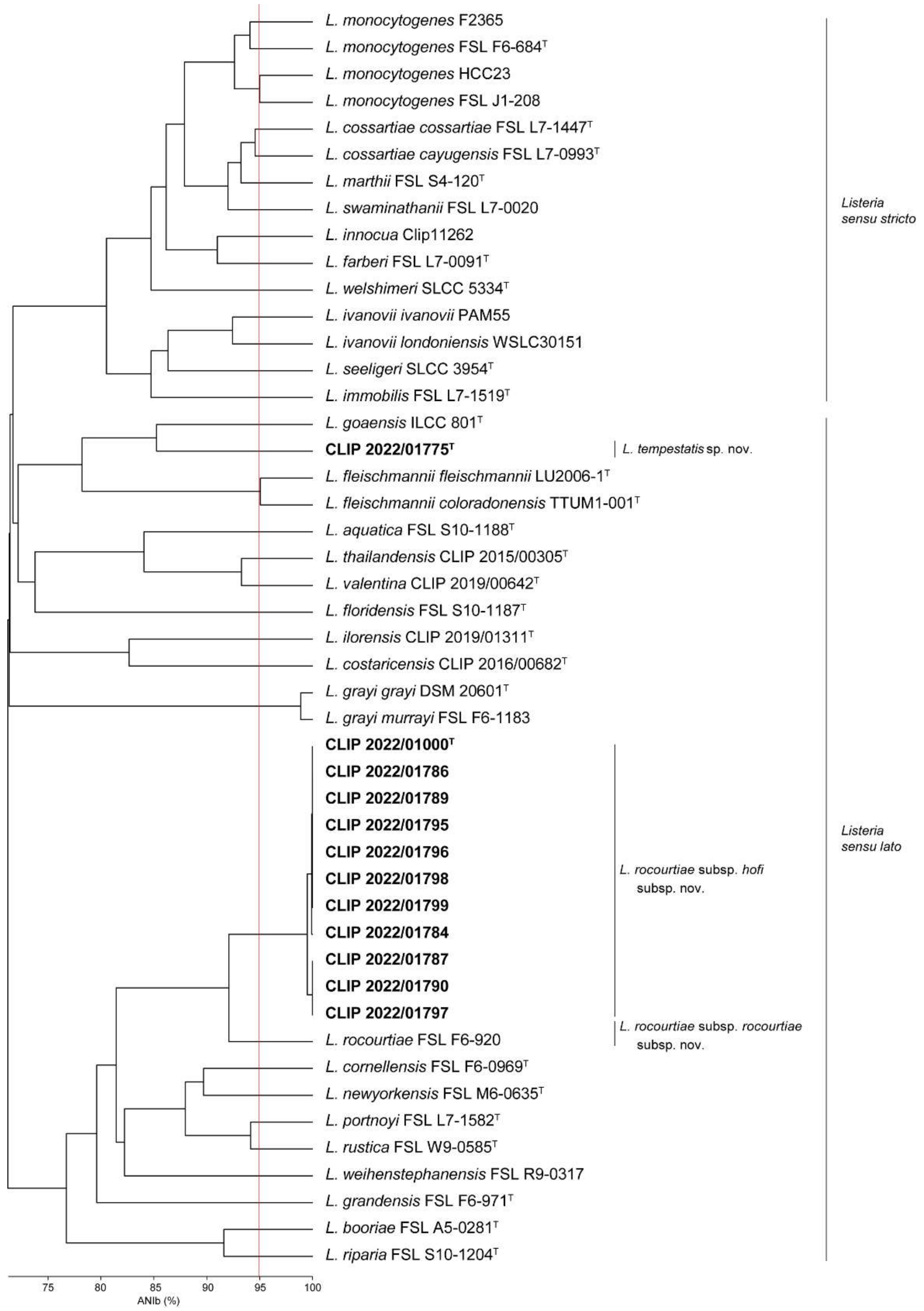

Supplementary Figure 2. UPGMA clustering based on the genomic average nucleotide difference by BLAST (ANIb). The vertical dashed bar represents the proposed 95% ANIb species cut-off that correlates with the 70% DNA-DNA hybridization threshold (41). Scale bar represents the percentage of similarity. *Listeria tempestatis* sp. nov. and *Listeria rocourtiae* subsp. *hofi* subsp. nov. are highlighted in bold.

Supplementary Table 1. Biochemical characteristics of species of the genus *Listeria* based on observations made in this study (highlighted in gray) and on previously published studies.

Characteristics	*Lmo*	*Lsw*	*Lcs*	*Lma*	*Lin*	*Lfr*	*Lw*	*Liv*	*Lse*	*Lim*	*Lgy*	*Lgo*	*Lte*	*Lfc*	*Laq*	*Lth*	*Lva*	*Lfo*	*Lil*	*Lco*	*Lho*	*Lro*	*Lcn*	*Lny*	*Lpo*	*Lru*	*Lgd*	*Lwp*	*Lbo*	*Lri*
Catalase	+	v	+	+	+	+	+	+	+	+	+	+	–	+	+	+	+	+	–	–	+	+	+	+	+	+	+	+	+	+
Motility	+	+	+	+	+	+	+	+	+	–	+	–	–	–	–	–	–	–	+	+	–	–	–	–	–	–	–	–	–	–
Voges–Proskauer	+	+	+	+	+	+	+	+	+	+	+	–	–	–	v	+	+	–	+	+	–	–	–	–	–	–	–	–	–	–
Nitrate reduction	–	–	–	–	–	–	–	–	–	–	v	–	+	+	+	+	+	–	–	–	+	+	+	+	+	+	+	+	+	+
Haemolysis	+	–	–	–	–	–	–	+	+	–	–	+ (α)	–	–	–	–	–	–	–	–	–	–	–	–	–	–	–	–	–	–
Phosphatidylinositol-specific phospholipase C (PI-PLC)	+	–	–	–	–	–	–	+	–	–	–	–	–	–	–	–	–	–	–	–	–	–	–	–	–	–	–	–	–	–
D-Arylamidase	–	–	–	–	+	+	v	v	+	+	+	–	–	–	–	–	–	–	–	–	–	–	–	–	–	–	–	–	–	–
α-Mannosidase	+	+	+	+	+	+	+	–	–	–	v	–	–	–	+	–	–	–	–	–	–	+	–	–	–	–	–	–	+	+
Acidification from:																														
D-Arabitol	+	+	+	+	+	+	+	+	+	+	+	(+)	–*	+	–	–*	–*	–	+	+	–	–	–	–	(+)	(+)	v	+	+	–
L-Arabinose	–	–	–	–	–	–	–	–	–	–	–	–	–	–	+	–	+	+	–	–	+	–	v	+	–	+	–	–	+	+
L-Fucose	–	–	–	–	–	–	–	–	–	–	–	–	–	–	–	–	–	–	–	+	–	–	–	–	–	–	–	–	–	–
D-Galactose	v	–	–	–	–	–	–	v	–	–	+	–	–	–	–	–	–	+	+	+	–	+	–	+	+	+	–	–	+	+
D-Glucose	v!	+	+	v!	v!	+	+	v!	+	+	+	+	+	+	+	+	+	+	+	+	+	+	+	+	+	+	+	+	+	+
Glucose 1-phosphate	–	–	–	–	–	–	–	v	–	–	–	–	–	–	–	–	–	–	–	–	–	–	–	–	–	–	–	–	–	–
Glycerol	v	v	+	–	+	–	+	+	+	v	v	(+)	–	+	v	(+)	(+)	–	–	–	v	+	v	+	–	–	–	+	(+)	v
Inositol	–	–	–	–	–	–	–	–	–	–	–	–	–	v	v	(+)	(+)	–	–	–	–	–	–	–	–	–	–	–	v!	v
Inulin	v!	–	–	–	v!	–	–	–	–	–	–	–	–	–	–	–	–	–	–	–	–	–	–	–	–	–	–	–	–	–
D-Lactose	+	–	+	+	+	+	+	+	+	v	+	+	–	+	–	–	–	+	+	+	–	+	(+)	+	(+)	+	–	v!	+	+
D-Lyxose	v	–	–	–	v	–	v	–	–	–	v	–	–	–	v	–	(+)	+	–	–	–	–	–	–	–	–	–	–	–	–
D-Maltose	+	+	+	+	+	+	+	+	+	v	+	+	+	+	–	–	–	+	+	+	(+)	(+)	+	+	–	+	+	+	+	+
D-Mannitol	–	–	–	–	–	–	–	–	–	–	+	–	–	v	–	–	–	–	–	–	–	+	–	+	+	+	–	+	+	v
D-Melezitose	v	–	–	–	v	–	v	v	v	v	–	–	–	v	–	–	–	–	–	–	–	–	–	–	–	–	–	–	–	–
D-Melibiose	v!	–	–	v	v	–	–	–	–	–	–	–	–	v	–	–	–	–	–	–	–	+	–	–	–	–	–	–	v!	v
Methyl α-D-glucopyranoside	+	+	+	+	+	+	+	+	+	+	+	+	+	+	–	–	–	+	+	+	+	+	+	+	+	+	+	+	+	+
Methyl α-D-mannopyranoside	v!	+	+	–	–	+	nd	–	nd	–	+	–	–	v	–	–	–	–	–	–	–	–	–	–	–	–	–	–	–	–
L-Rhamnose	+	–	–	–	v	+	v	–	–	–	–	+	+	+	+	+	+	+	–	–	–	+	–	v	+	+	–	+	+	+
D-Ribose	–	–	v	–	–	–	–	+	–	+	+	–	+	+	+	+	+	–	+	+	+	+	+	+	–	–	+	–	v	v
D-Saccharose (sucrose)	v!	–	–	–	+	–	+	+	+	v	–	–	+	v	–	–	–	–	–	+	–	–	–	–	–	–	–	–	–	–
L-Sorbose	v!	–	–	v!	v!	–	–	v!	–	–	v!	–	–	v	–	(+)	–	–	–	–	–	–	–	–	–	–	–	–	–	–
D-Tagatose	–	–	–	–	–	–	+	–	–	–	–	–	–	–	+	+	v!	–	–	–	–	–	–	–	–	–	–	–	v!	–
D-Turanose	–	–	–	+	v	–	–	–	–	–	–	–	–	v	–	–	–	–	–	–	–	–	–	–	–	–	–	–	–	–
D-Xylose	–	–	–	–	–	–	+	+	+	+	–	+	+	+	+	+	+	+	+	+	+	+	+	+	+	+	+	+	+	+

All species/strains are positive for aesculin and acid production from N-acetylglucosamine, amygdalin, arbutin, D-cellobiose, D-fructose, D-mannose and salicin. All species/strains are negative for nitrite reduction and acid production from D-adonitol, D-arabinose, glycogen, methyl β-D-xylopyranoside, potassium 2-ketogluconate, and D-raffinose.

Species: *Lmo, L. monocytogenes; Lsw, L. swaminathanii; Lcs, L. cossartiae; Lma, L. marthii; Lin, L. innocua; Lfr, L. farberi; Lws, L. welshimeri; Liv, L. ivanovii; Lim, L. immobilis; Lse, L. seeligeri; Lgy, L. grayi; Lth, L. thailandensis; Lva, L. valentina; Laq, L. aquatica; Lfo, L. floridensis; Lfc, L. fleischmannii; Lgo, L. goaensis; Lil, L. ilorinensis*; *Lco, L. costaricensis; Lru, L. rustica; Lpo, L. portnoyi; Lcn, L. cornellensis; Lny, L. newyorkensis; Lro, L. rocourtiae* subsp. *rocourtiae* subsp. mov.*; Lwp, L. weihenstephanensis; Lgd, L. grandensis; Lbo, L. booriae; Lri, L. riparia; Lho, L. rocourtiae* subsp. *hofi* subsp. nov. (this study)*; Lte, L. tempestatis* sp. nov. (this study).Notation: +, positive; (+), weakly positive; −, negative; v, variable between strains and/or replicates; v!, variable between studies (possibly due to differences in incubation times and temperatures); *, based on API 50 CH (positive on API Listeria); nd, not determined.
